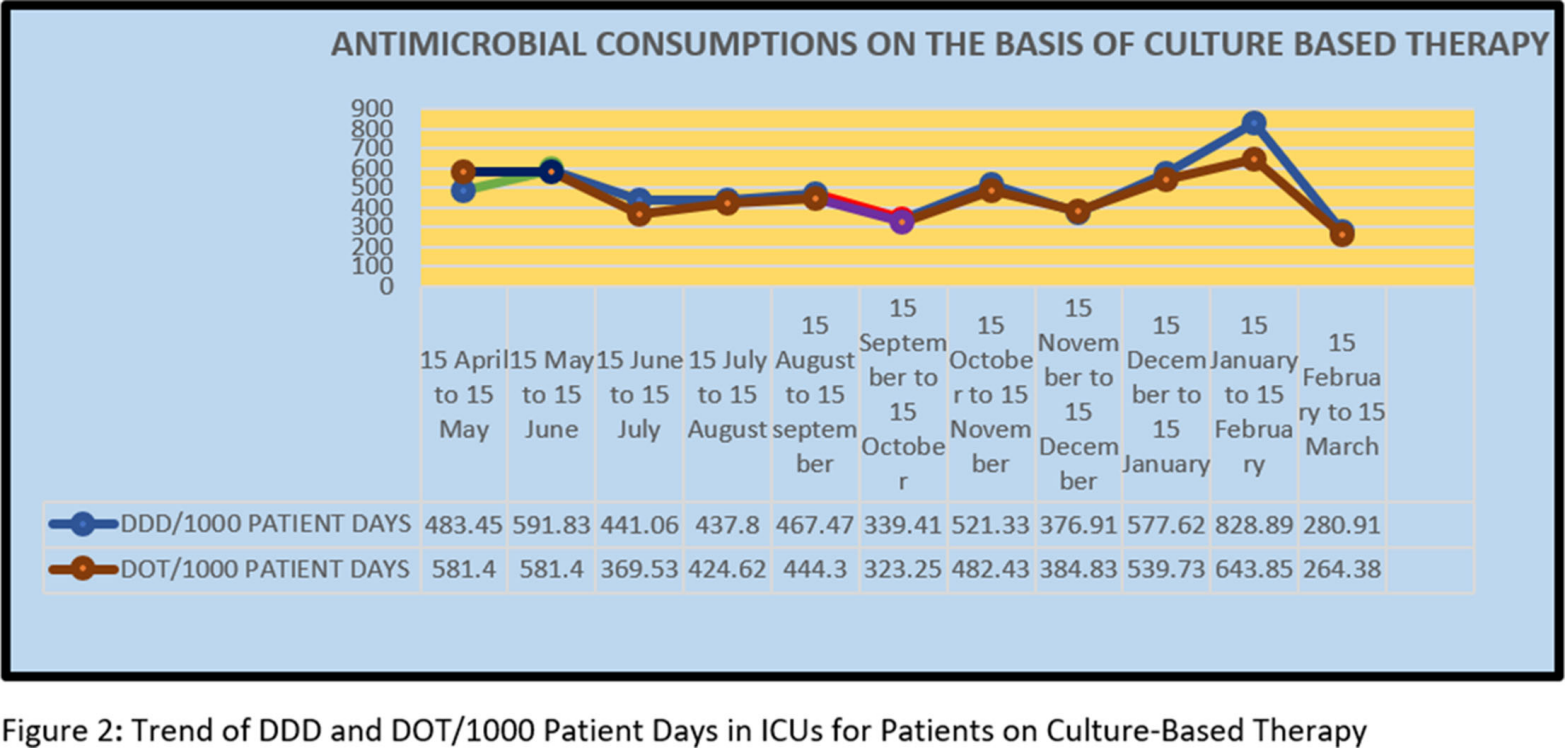# Prospective Audit for Antimicrobial Use and Stewardship Practices in Intensive Care Unit at a Tertiary-Care Center in India

**DOI:** 10.1017/ash.2021.74

**Published:** 2021-07-29

**Authors:** Parul Singh, Purva Mathur, Kamini Walia, Anjan Trikha

## Abstract

**Background:** Antimicrobial decision making in the ICU is challenging. Injudicious use of antimicrobials contributes to the development of resistant pathogens and drug-related adverse events. However, inadequate antimicrobial therapy is associated with mortality in critically ill patients. Antimicrobial stewardship programs are increasingly being implemented to improve prescribing. **Methods:** This prospective study was conducted over 11 months, during which the pharmacist used a standardized survey form to collect data on antibiotic use. Evaluation of antimicrobial use and stewardship practices in a 12-bed polytrauma ICU and a 20-bed neurosurgery ICU of the 248-bed AIIMS Trauma Center in Delhi, India. Antimicrobial consumption was measured using WHO-recommended defined daily dose (DDD) of given antimicrobials and days of therapy (DOT). **Results:** Antibiotics were ranked by frequency of use over the 11-month period based on empirical therapy and culture-based therapy. The 11-month DDD and DOT averages when empiric antibiotics were used were 532 of 1,000 patient days and 484 per 1,000 patient days, respectively (Figure 1). When cultures were available, DDD was 486 per 1,000 patient days and DOT was 442 per 1,000 patient days (Figure). **Conclusions:** The quantity and frequency of antibiotics used in the ICUs allowed the AMSP to identify areas to optimize antibiotic use such as educational initiatives, early specimen collection, and audit and feedback opportunities.

**Funding:** No

**Disclosures:** None

**Figure 1.**
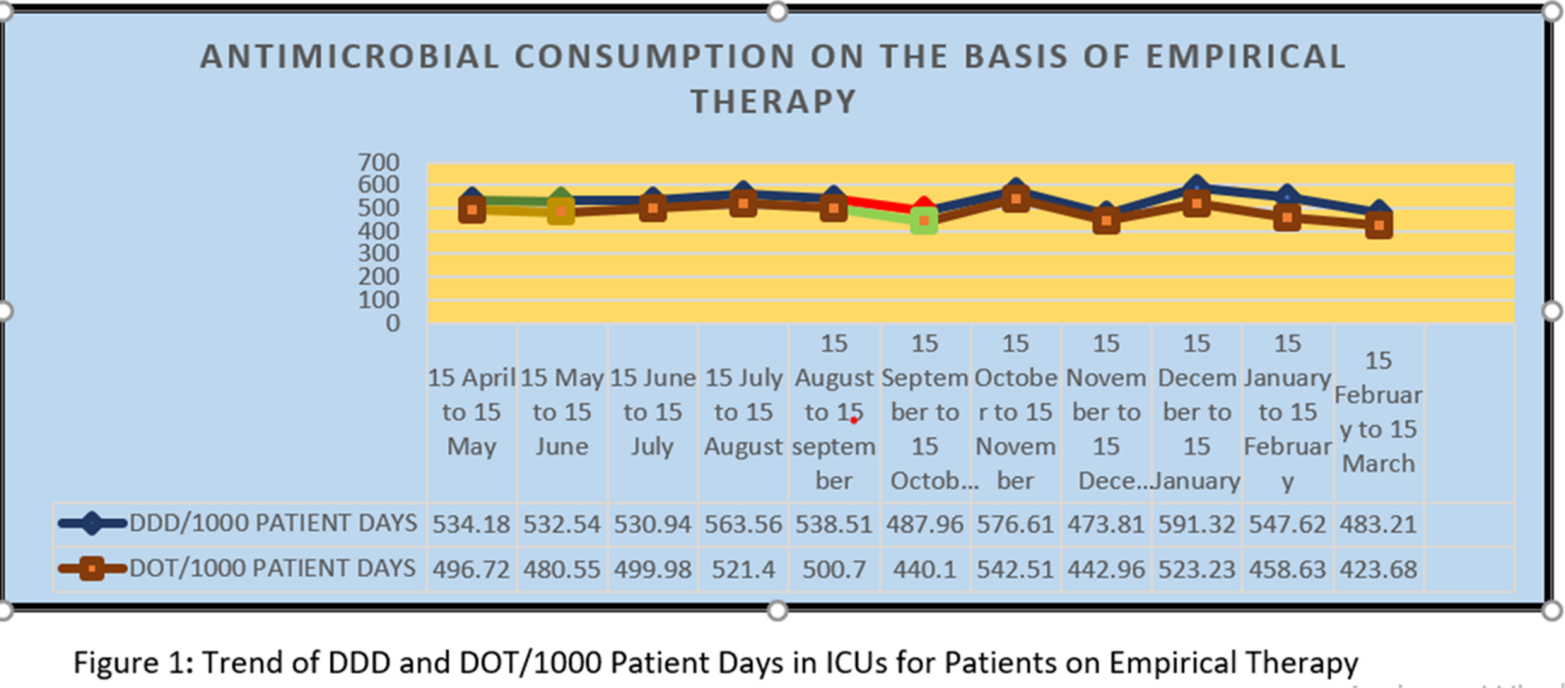

**Figure 2.**